# Delayed solitary mediastinal lymph node metastasis from a benign meningothelial meningioma six years after surgical resection of an intracranial tumor

**DOI:** 10.1111/1759-7714.13996

**Published:** 2021-05-07

**Authors:** Jae Wan Jung, Chul Park

**Affiliations:** ^1^ Division of Pulmonary Medicine, Department of Internal Medicine Wonkwang University Hospital Iksan South Korea

**Keywords:** lymph nodes, mediastinal neoplasm, meningioma, neoplasm metastasis

## Abstract

Meningiomas are common intracranial neoplasms with benign features, and extracranial metastases are very rare. There have been no previous reports of solitary mediastinal lymph node metastasis from benign meningiomas without pulmonary lesions. Here, we present a case of an 82‐year‐old female who visited our department for mediastinal lymphadenopathy with a history of meningioma treated with total surgical resection six years prior. Endobronchial ultrasound‐guided transbronchial needle aspiration of the left lower paratracheal lymph node revealed a benign meningothelial meningioma. In patients with a history of meningioma, extracranial metastasis should be considered in the differential diagnosis of mediastinal lymphadenopathy.

## Graphical abstract

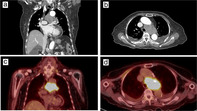

## INTRODUCTION

Meningiomas are common intracranial neoplasms and tend to have a benign clinical course.^1^ Extracranial metastases from meningiomas are an exceedingly uncommon event found in <0.1%–0.2% of patients, many of whom have advanced disseminated disease.^2^

Here, we report the first case of a benign meningothelial meningioma with solitary mediastinal lymph node metastasis in a patient without lung metastasis. Mediastinal lymph node metastasis was detected six years after surgical resection of the intracranial meningioma and diagnosed by endobronchial ultrasound‐guided transbronchial needle aspiration (EBUS‐TBNA).

## CASE REPORT

An 82‐year‐old woman was referred to our department with mediastinal lymphadenopathy, which was detected incidentally on workup for a rib fracture. The patient had a history of a meningioma and had been treated with total surgical resection at another hospital six years prior. Medical comorbidities included hypertension and diabetes mellitus. She had no family history of malignancy.

**FIGURE 1:**
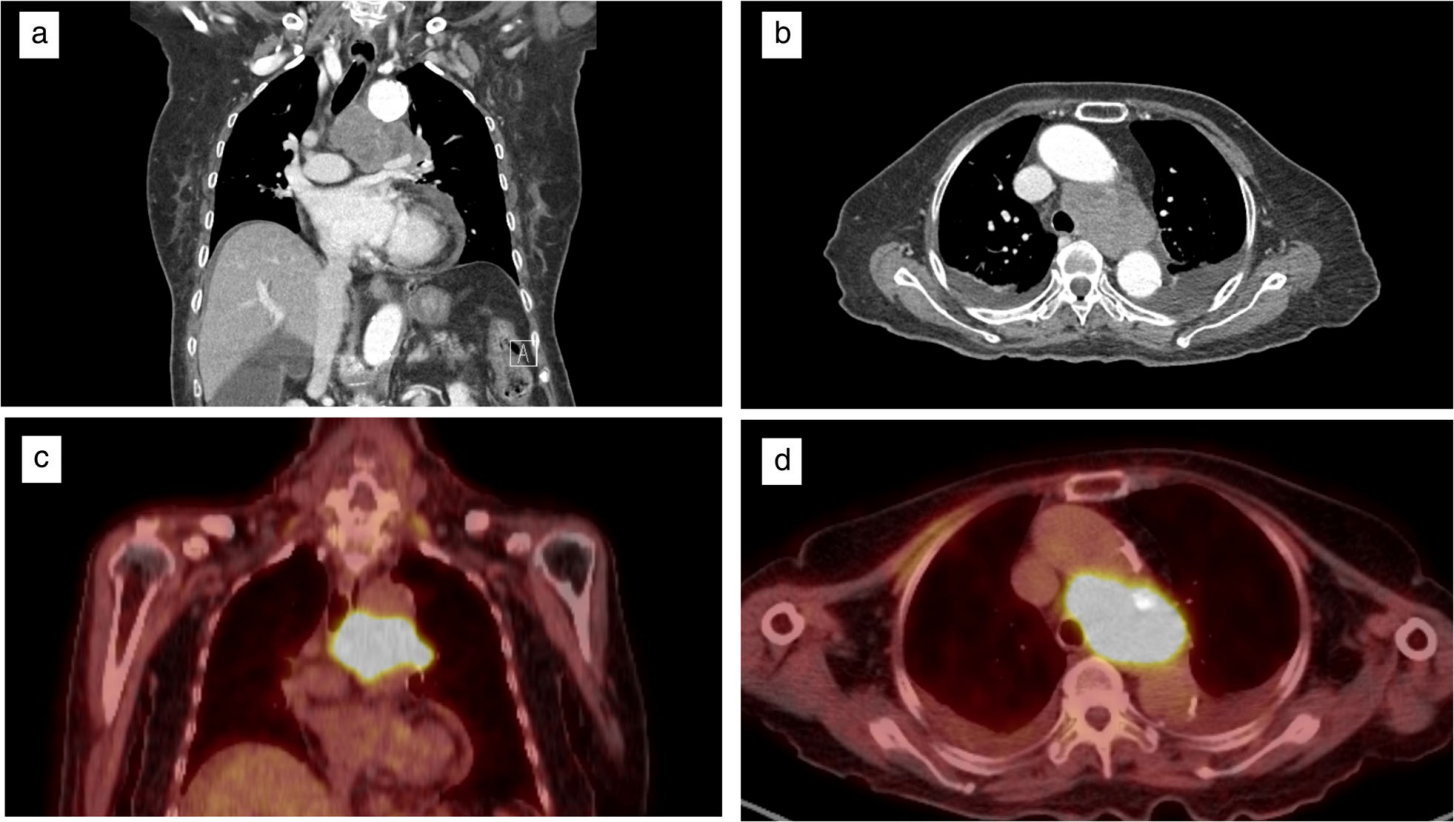
(a) Contrast‐enhanced computed tomography (CT) revealed a bulky lymphadenopathy in the left lower paratracheal area compressing the lower trachea and left main pulmonary artery. (b) On an axial section of the enhanced CT scan, a mediastinal mass compressing the left main bronchus was observed. (c), (d) 18F‐fluorodeoxyglucose‐positron emission tomography (FDG‐PET) showed high uptake of FDG with a maximal standardized uptake value of 9.2 by the tumor

**FIGURE 2:**
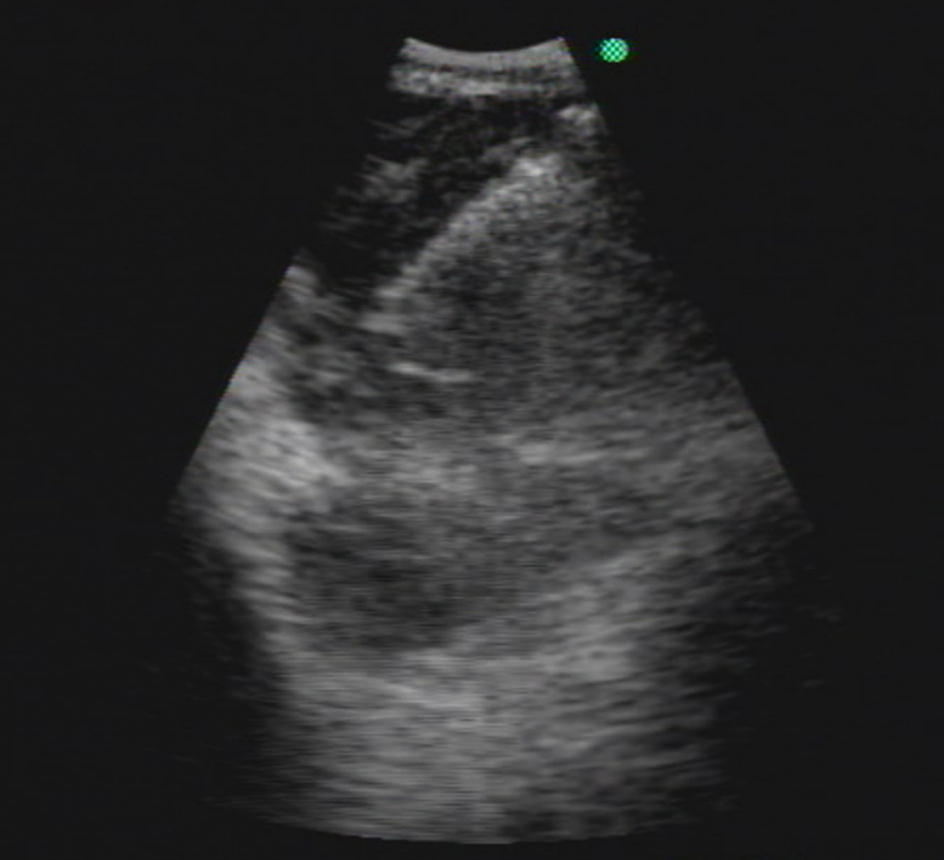
Endobronchial ultrasonography‐guided transbronchial needle aspiration (EBUS‐TBNA)

**FIGURE 3:**
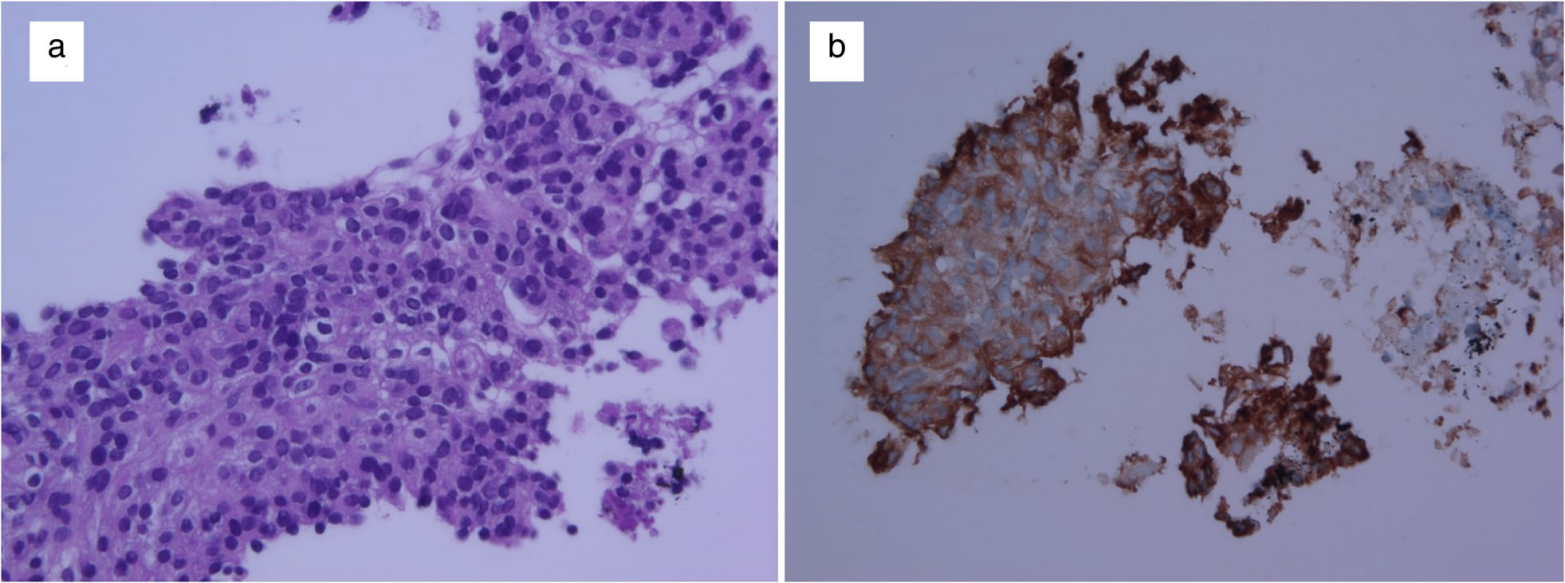
(a) Tumor cells had round uniform nuclei with indistinct cell membranes (hematoxylin & eosin [H&E], ×400). (b) Immunohistochemical staining revealed immunoreactivity for vimentin (vimentin, ×400)

Laboratory data were all within normal range. Contrast‐enhanced computed tomography (CT) revealed a bulky lymphadenopathy, 75 mm in diameter, located in the left lower paratracheal to the left hilar area. The mass was compressing the lower trachea, left main bronchus, and left main pulmonary artery (Figure 1(a) and 1(b)). 18F‐fluorodeoxyglucose‐positron emission tomography (FDG‐PET) revealed high uptake of FDG with a maximal standardized uptake value of 9.2 (Figure 1(c) and 1(d)). Endobronchial ultrasound (EBUS) demonstrated an ill‐defined heterogeneous mass in the left lower paratracheal area. The patient underwent EBUS‐TBNA (Figure 2). Histological analysis revealed monotonous tumor cells with round‐to‐ovoid nuclei and indistinct cell membranes (Figure 3(a)). The tumor was low grade and had a low proliferation index. Immunohistochemical staining was negative for thyroid transcription factor‐1 and pan‐cytokeratin, and positive for vimentin (Figure 3(b)), findings compatible with meningothelial meningioma according to WHO classification (Table 1). The chest CT image showed a small amount of bilateral pleural effusion; however, we did not conduct oncological search data for pleural effusion analysis. First, the standardized uptake value (SUV) at the pleural effusion site was 1.4. Second, we determined that both pleural effusions were of systemic origin because the patient was malnourished due to insufficient oral intake. Thus, we determined the possibility of malignant effusion was low. The patient was asymptomatic with regard to metastasis, and the decision was made to proceed conservatively with observation and annual imaging.

**TABLE 1:** WHO classification of meningiomas

WHO grade 1	WHO grade 2	WHO grade 3
Meningothelial	Chordoid	Papillary
Fibrous	Clear cell	Rhabdoid
Transitional	Atypical	Anaplastic (malignant)
Psammomatous	Increased mitoses 4–19 mitoses/10 HPF	Overtly malignant cytology (resembling carcinoma, melanoma, high‐grade sarcoma)
Angiomatous		
Microcytic	Brain invasion	
Secretory	At least three of the following features: Increased cellularity Small cells with high nuclear‐to‐cytoplasmic ratio Prominent nucleoli Sheeting Foci of spontaneous necrosis	
Lymphoplasmacyte‐rich		Markedly increased mitoses ≥ 20 mitoses/10 HPF
Metaplastic		


## DISCUSSION

Most meningiomas behave in a benign course, but can sometimes display malignant behavior, such as metastasis. Distant metastases have been reported sporadically in case reports of atypical and anaplastic meningiomas, but have rarely been reported for benign meningiomas.^3^ Metastasis to the mediastinal lymph nodes is usually found concurrently with pulmonary metastasis. Mediastinal lymph node metastasis from a meningioma without pulmonary metastasis is very rare; we found only two cases while searching Medline.^4^, ^5^ One case involved a mediastinal metastasis from an intracranial meningioma, similar to the case presented here, while the second case involved metastasis from an intramedullary spinal meningioma of the thoracic vertebra. However, both previous cases involved an atypical meningioma, unlike our case. This is the first case report in the literature of a solitary mediastinal metastasis from a benign meningioma.

The WHO classification of CNS tumors divides meningiomas into three major groups: grade I (benign), grade II (atypical), and grade III (anaplastic) (Table 1).^6^ According to WHO criteria, the grade of the tumor is the most important predictive factor for recurrence and metastasis.^7^ However, these features are not essential for the occurrence of extracranial metastasis, and any histologically benign meningioma has the potential to metastasize.^7^ This is evident in our case of mediastinal metastasis from a benign meningothelial meningioma. Despite lack of mitotic activity, a low proliferation index, and no local tumor recurrence, our patient presented with solitary mediastinal metastasis.

Extracranial metastases of meningioma have been detected in the lungs (60%), abdomen and liver (34%), cervical lymph nodes (18%), long bones, pelvis, and skull (11%), pleura (9%), vertebrae (7%), and mediastinum (5%).^7^ Meningiomas may disseminate through hematogenous, lymphatic, or cerebrospinal fluid (CSF) routes.^8^ Hematogenous metastasis via the jugular vein may be the most frequent pathway for metastatic dissemination of meningiomas.^2^ The passage of tumor cells into venous channels allows spread through the right blood circulation into the lungs, liver, and other organs.^9^ However, meningiomas can also metastasize via the paravertebral venous plexus.^2^ The paravertebral venous plexus has multiple connections with pelvic and thoracic vessels to intraspinal veins. Meningiomas can also metastasize through lymphatic vessels or CSF.

In our case, the route of metastasis is of particular importance, and we developed several hypotheses to explain it. First, the tumor could have spread via the paravertebral venous plexus. Cases of solitary mediastinal lymph node metastasis in malignant extrathoracic tumors via the paravertebral venous plexus have previously been reported.^10^, ^11^ Next, the solitary mediastinal lymph node could function as a catchment area for an early small pulmonary metastasis. The primary pulmonary metastasis was so small that only the lymph node was identified by diagnostic imaging. Metastasis through CSF or the lymphatic pathway is unlikely to have occurred in our patient.

No consensus on the optimal treatment for extracranial metastatic meningioma has been established to date. Although there are reports of curative metastatectomies for meningiomas, the long‐term prognosis in this rare patient population is not well defined.^12^ Chemotherapy is of limited efficacy in meningioma, and systemic therapy is of limited or no benefit.^9^ If asymptomatic, we advocate a conservative “watch‐and‐wait” strategy involving regular imaging.

In conclusion, this is the first reported case of a solitary mediastinal metastasis from a benign meningioma several years after initial tumor resection. This case highlights the aggressive potential of meningiomas, which are typically considered benign tumors, and emphasizes the need to consider metastatic meningioma in the workup of mediastinal masses in patients with a history of meningioma.

## CONFLICT OF INTEREST

The authors declare that there are no conflicts of interest.
